# Bidirectional associations between maternal depressive symptomatology and parenting: moderating effects of breastfeeding and partner support

**DOI:** 10.1186/s40748-026-00279-2

**Published:** 2026-08-05

**Authors:** Élisabeth Morin, Jean R. Séguin, Sophie Parent, Catherine M. Herba, Lise Dubois, Gina Muckle, Michel Boivin, William D. Fraser, Natalie Castellanos Ryan

**Affiliations:** 1 Research Centre, CHU Ste-Justine, Montreal, Canada; 2 Psychoeducation Department, University of Montreal, Montreal, Canada; 3 Psychiatry and Addictology Department, University of Montreal, Montreal, Canada; 4 Psychology Department, University of Quebec at Montreal, Montreal, Canada; 5 Centre Hospitalier Universitaire de Sherbrooke (CHUS) Research Center, Sherbrooke, Canada; 6 Department of Obstetrics and Gynecology, University of Sherbrooke, Sherbrooke, Canada; 7 School of Epidemiology and Public Health, University of Ottawa, Ottawa, ON Canada; 8 Research Centre, School of Psychology, Centre Hospitalier Universitaire de Québec, Laval University, Quebec City, Canada; 9 Department of Psychology, University Laval, Quebec City, Canada

**Keywords:** Postpartum depression, Parenting, Longitudinal, Breastfeeding, Partner support

## Abstract

Postpartum depression and parenting both play a role in shaping children’s future behavior. However, there is limited understanding of the interaction and the evolving relationship between depressive symptoms and parenting practices. This study aimed to investigate the bidirectional associations between maternal depressive symptomatology and parenting (maternal self-efficacy, maternal warmth, and maternal hostile-reactive behaviors) in the postpartum period and to assess the moderating effect of breastfeeding and partner support on these associations. Latent growth curve and multigroup analyses were estimated in a sample of 1551 mothers of the 3D pregnancy cohort. Maternal depressive symptomatology and parenting practices were concurrently associated. Bidirectional associations were found between maternal depressive symptomatology and maternal self-efficacy, but not for warmth or hostile-reactive behaviors. Mothers with high perceived partner support showed a significant association between increased maternal warmth and lower depressive symptoms at 24 months. In contrast, mothers with lower perceived partner support demonstrated stronger associations between early hostile-reactive parenting and later depressive symptoms, as well as a greater impact of declining self-efficacy on maternal depressive symptoms. Breastfeeding showed limited moderating effects, with no significant buffering impact on the associations between depressive symptoms and parenting practices. These findings highlight the protective role of partner support in mitigating the adverse effects of depressive symptoms on parenting and vice versa, emphasizing the need for interventions that strengthen maternal self-efficacy and partner involvement to promote maternal mental health.

## Introduction

Pregnancy, childbirth, and the transition into motherhood represent profound milestones in a woman’s life, often bringing a mix of joy, anticipation, and challenges. It is a period of increased vulnerability to emotional disorders, particularly postpartum depression (PPD). It affects between 10% and 20% of women globally, with 25% of new mothers in Canada reporting symptoms [1–3]; Statistics Canada, [4, 5]. However, the true prevalence may be even higher due to barriers such as stigma and undetected cases. As one of the most common perinatal disorders, PPD not only undermines maternal well-being but is associated with less optimal child development, with intergenerational effects. Additionally, it incurs significant familial and societal costs [1, 6, 7]; Statistics Canada, [4].

### Maternal depression and parenting behaviors

Notably, PPD has been associated with poorer parenting strategies, such as those characterized by low self-efficacy, less healthy practices, less closeness, warmth and sensitivity [8]. The Integrative Model of Risk for Psychopathology in the Children of Depressed Mothers, proposed by Goodman and Gotlib [9], outlines four key mechanisms through which maternal depression may be related to adverse child outcomes: genetic and epigenetic factors, exposure to stressors, prenatal influences, and parenting behaviors. Parenting is a key pathway in this model, with evidence supporting its role in mediating associations between maternal depression and child outcomes, even when genetic factors are considered [10]. This developmental model assumes longitudinal processes, such as the intergenerational transmission of risk and the gradual unfolding of mechanisms like parenting over time. However, a significant proportion of the empirical work testing the model’s hypotheses has relied on cross-sectional data, unidirectional designs, and small cohorts, which limits the ability to confirm the temporal pathway proposed in the model.

Building on this model, recent longitudinal studies have explored how maternal depressive symptoms (MDS) are associated with parenting in early childhood. For example, Hentges et al., [11], in a longitudinal study of a large cohort, suggested that hostile parenting may mediate the transmission of MDS to children internalizing problems, with assessments conducted from 25 weeks of gestation to 5 years of age. Errazuriz Arellano et al. [12] longitudinal study of 199 mothers and their children (aged 3 to 6 years) found that higher MDS were associated with parenting characterized by increased overreactivity and laxness, and reduced warmth by age 6. Similarly, in another longitudinal study, Mitchell et al. [13] reported that higher maternal prenatal depressive symptoms predicted lower warmth at 4 months postpartum, although changes in depressive symptoms were not related to changes in warm responsiveness over time. Guyon-Harris et al. [14] observed that mothers with high depressive affect and physical symptoms (e.g., sleep and eating difficulties, low energy, trouble initiating tasks, and increased agitation) were at greater risk of harsh parenting at age 2 and disengaged parenting at age 3. Notably, no differences in positive parenting were found across depression profiles in this study. While these studies were limited to specific populations, mothers of children with behavioral problems [12], predominantly single mothers [13], and a low-income community sample [14], they provided support for Goodman and Gotlib’s [9] model, by highlighting associations between MDS and parenting. Importantly, Errazuriz Arellano et al. [12] also suggested that associations between MDS and parenting may be bidirectional. Supporting this idea, recent studies and meta-analyses have highlighted the transactional and bidirectional nature of this relationship. Specifically, early MDS may be associated with less effective parenting, which then may exacerbate MDS, creating a feedback loop that perpetuates the cycle, a dynamic that may be further amplified by children’s behavioral and emotional responses to less effective parenting, such as dysregulation or increased negativity [15, 16]. This emphasizes the need to further investigate how MDS and parenting may be dynamically interrelated over time [17].

Hails et al., [10] used a cross-lagged panel design to explore transactional relations and found that parent–child coercion at age two years predicted higher MDS at age three, which were then associated with increased coercion at age four. Barboza and Schiamberg, [18] using longitudinal data from the third pregnancy trimester to 3 years, suggested that low parental self-efficacy might exacerbate depressive symptoms, potentially creating a negative feedback loop. Both studies were conducted with specific groups: low-income families [10] and young, unmarried mothers of color, whose children were considered at risk of socioemotional problems [18]. This limits the generalizability of their findings to other demographic groups where the dynamics between MDS and parenting may differ. These sample-specific limitations highlight the need for research that explores these relationships in more socioeconomically and demographically varied populations. Goodman et al. [17] meta-analytic review of 35 longitudinal studies involving children from 0 to 18 years estimated the pooled effects of both early maternal depression on later parent self-efficacy and parent self-efficacy on later depression, with nearly identical effect sizes (d = − 0.21 and − 0.22, respectively). Of the 35 longitudinal studies included in the meta-analysis, 20 examined only a unidirectional relationship. Despite this limitation, these findings underscore possible bidirectional associations between MDS and maternal self-efficacy.

Despite recent methodological advancements facilitating the examination of transactional and bidirectional associations, much of the literature has relied on single time-point measures of parenting, which fail to capture its dynamic nature and the ways it may evolve over time [17]. To capture these complex and dynamic changes and relations across time, analytic approaches that can model intra-individual changes and account for bidirectional and transactional associations are essential. Latent growth curve and cross-lag panel models are valuable approaches for capturing change over time and the examination of bidirectional effects, respectively. These models allow for the examination of whether early MDS may predict changes in later parenting, and how early parenting may predict later MDS, while also accounting for their stability. Such an approach could address existing gaps in the literature, particularly the reliance on single time-point measures, and contribute to a more comprehensive understanding of these complex relationships.

### Maternal depressive symptoms and parenting: roles of breastfeeding and partner support

Moderating factors, such as breastfeeding and partner support, are often highlighted in the depression-parenting association [8, 19, 20]. Although breastfeeding has been cited as a protective factor in maternal mental health and parenting outcomes [8, 20, 21], these effects are thought to operate through multiple pathways, including hormonal mechanisms, particularly the release of oxytocin during nursing, which may reduce stress reactivity and promote positive affect, as well as increased opportunities for maternal responsiveness and the development of a sense of competence in the caregiving role [22]. At the same time, breastfeeding experiences are not uniformly positive; difficulties with initiation or maintenance may heighten maternal distress, particularly among mothers with pre-existing depressive symptoms [23, 24]. Its potential moderating role in the association between MDS and parenting remains largely unexplored. Some evidence suggests that breastfeeding may buffer the impact of MDS, with longer durations linked to greater attachment security and lower postpartum depression risk [25, 26]. A meta-analysis by Xia et al. [27] further supports the idea that breastfeeding beyond the first month reduces depressive symptoms, yet few studies have tested whether it moderates the depression-parenting link. Sliwerski et al. [20] highlight this gap, calling for longitudinal research to examine whether breastfeeding mitigates the effects of MDS on parenting behaviors.

Partner support may also buffer the effects of MDS on parenting. Some studies suggest a protective role, with lower MDS at six weeks postpartum when partner support is high [28] and reduced postpartum depression risk overall [29]. However, other research indicates that this association varies depending on context. Brazeau et al. [30] found that general emotional support moderated associations between MDS and maternal self-efficacy, yet Taraban et al. [31] found that this association was stronger in the context of high marital quality and high social support. They suggest that in a high-quality marriage, fewer risk factors may be present, making the association between maternal depression and parenting more salient. The literature thus suggests that breastfeeding and partner support may buffer links between MDS and parenting, whereby depressive symptoms may be more strongly linked to parenting in the context of lower partner support or shorter breastfeeding duration.

### The current study

This research builds on Goodman and Gotlib’s [9] model by expanding our understanding of how MDS and parenting dimensions may be bidirectionally and transactionally associated over time, and how breastfeeding and/or partner support could buffer these associations. Few studies have addressed these dynamics or their potential moderators. This study addresses this gap by focusing on three key parenting dimensions: self-efficacy, warmth, and hostile-reactive behaviors from three months to two years of age, and modelling these longitudinally using latent growth curve and cross-lag panel models. We hypothesized bidirectional links between MDS and parenting dimensions across time would be found: 1a) higher maternal depressive symptoms at three months would predict lower maternal warmth and self-efficacy, and higher hostile-reactive behavior from three months to 2 years, and 1b) lower maternal warmth and self-efficacy, and higher hostile-reactive behavior at three months would predict higher maternal depressive symptoms at 2 years. Specifically, we expect that maternal depressive symptoms and parenting dimensions may predict each other over time. We will further examine the moderating role of breastfeeding and partner support, hypothesizing that 2) breastfeeding and high perceived partner support will attenuate the association between maternal depressive symptoms and parenting dimensions over time. Clarifying the bidirectional and transactional dynamics between postpartum depressive symptoms and parenting, along with the moderating effects of breastfeeding and partner support, could provide valuable insight for developing effective interventions that promote maternal and child health.

## Materials and methods

### Participants and procedure

Participants were drawn from the 3D (Design, Develop, Discover) Study, a pregnancy cohort study (ClinicalTrials.gov #NCT03113331, registered April 13, 2017). The 3D study aimed to investigate relationships between prenatal adverse exposures, birth outcomes, and subsequent health outcomes in children [32]. Over a three-year period, 2,366 pregnant participants and their partners were recruited in the first trimester of pregnancy (8–14 weeks), at participating university hospitals and clinical sites in Québec, Canada. Eligible participants were those who could communicate in French or English and consented to participate. Data were initially collected during the first, second, and third trimesters, and following delivery. For live births, postnatal visits took place at 3 months, 12 months, and 24 months. Due to various factors such as attrition, incomplete data, or withdrawal from the study, the final sample consisted of 1551 participants and children from the 3D cohort study who completed questionnaires and measurements up to 24 months. At recruitment, the average age of pregnant participants was 31 years (range 17 to 46 years). Approximately 53% of the sample reported a pre-tax annual income of 80,000 CAD or higher, which slightly exceeds the average income level during that period (Statistics Canada, [33]). Additional descriptive statistics of demographic and study variables are provided in Table 1. The sample used for analyses (*n* = 1551) did not differ significantly from the original 2366 sample on age, ethnicity, marital status, education, income and depression. The multicenter study was approved by the central site (CHUSJ research ethics committee) and institutional ethics committees at all participating sites.

**Table 1:** Demographic characteristics of sample (*n* = 1551)

Variable	Category	% (Mean, SD)
Maternal age		(31, 4.4)
Marital status	Partnered	95.2
	Single	4.8
Ethnicity		
	Canadian	67.2
	African	9.7
	European	8.6
	Mexican or South American	5.7
	Asian	4.9
	Caribbean	3.3
	American	0.5
	Oceanian	0.1
Education		
	High school or less	8.5
	College/technical/CEGEP	26.6
	Bachelor’s or higher	64.9
Family income		
	$80,000 CAD or less	47.2
	$80,000 CAD or higher	52.8
MDS 3 mo.		(5.95, 4.22)
MDS 24 mo.		(9.79, 3.55)
Mother’s SE 3 mo.		(8.55, 0.98)
Mother’s SE 12 mo.		(8.52, 0.97)
Mother’s SE 24 mo.		(8.36, 1.04)
Mother’s HR 3 mo.		(1.16, 1.26)
Mother’s HR 12 mo.		(2.22, 1.39)
Mother’s HR 24 mo.		(3.02, 1.76)
Mother’s WR 3 mo.		(9.39, 0.73)
Mother’s WR 12 mo.		(9.29, 0.78)
Mother’s WR 24 mo.		(9.14, 0.86)
Partner support		(7.69, 1.64)
Breastfeeding		
	≤ than 6 months	32.2
	≥ than 6 months	67.8

MDS = Maternal depressive symptomatology; SE = Mother’s self-efficacy; HR=Mother’s hostile-reactive behavior; WR=Mother’s warmth

### Missing data

Missing data among the 1551 participants included in the analyses was mainly from loss to follow up at 24 months, where 46% of the data were missing. Participants with missing data were compared to those with complete data, revealing that those without missing data were more likely to complete graduate studies (*χ*^2^(1) = 20.40, *p* < 0.001), be born in Canada (*χ*^2^(1) = 31.47, *p* < 0.001) and report a household income of more than 100 000$ (*χ*^2^(1) = 17.45, *p* < 0.001). Child sex and maternal depressive symptoms were not associated with missing data. Full information maximum likelihood (FIML) was used to account for missing data in analyses.

### Measures

#### Maternal symptoms of depression

MDS was measured using the CESD-10 [34] a short-form adaptation of Radloff’s [35] 20-item Center for Epidemiologic Studies Depression Scale. Postnatal data were collected at three, 12, and 24 months. Each item was rated on a three-point Likert scale (0 = rarely/never to 3 = most/all the time), with total scores ranging from 0 to 24. The questionnaire had good consistency at three months (α = 0.79) and 24 months (α = 0.63).

#### Parenting practices

The Parental Cognitions and Conduct Toward the Infant Scale (PACOTIS) [36], a self-reported instrument, was used to measure parents’ perceptions of their parenting practices at three, 12 and 24 months. The original version comprises 32 items grouped into five subscales: *parental self-efficacy*,* parental warmth perceived parental impact*,* parental hostile-reactive behaviors*,* and parental overprotection*. For this study, three of the five subscales were used: *parental self-efficacy*,* parental warmth*,* and parental hostile-reactive behaviors.* Each statement was measured using a Likert scale ranging from 0 (not at all what I think, feel, or do) to 10 (exactly what I think, feel, or do). The parental warmth subscale demonstrated good internal consistency across all time points, with Cronbach’s alpha values of 0.73 at three months and 0.75 at both 12 and 24 months. Similarly, the parental self-efficacy subscale showed reliability, ranging from 0.68 at three months to 0.76 at 12 months and 0.73 at 24 months. In contrast, the parental hostile-reactive behaviors subscale exhibited more variability, with good consistency at three months (α = 0.69), peaking at 12 months (α = 0.81), and decreasing at 24 months (α = 0.57).

#### Breastfeeding

Face-to-face interviews with the mother, conducted during postnatal visits when the children were aged three and 12 months, yielded information about the start, duration and exclusivity of breastfeeding. Based on data collected up to 12 months, categorical variables were derived for breastfeeding status at 6 months concerning (1) the total duration of breastfeeding (i.e., the entire period during which the child received breast milk, whether or not other liquids or solids were offered); (2) the duration of exclusive breastfeeding (i.e., the period during which the child was fed only breast milk, to the exclusion of all other liquids (e.g., water, formula, etc.) or solid foods) [37]. Despite well-documented health benefits, global rates of exclusive breastfeeding remain low [38–40]. As a result, focusing solely on exclusive breastfeeding may not fully capture the broader patterns and realities of infant feeding practices. For this study, the first variable, representing the total duration of breastfeeding at six months, was used. Mothers were categorized into two groups: those who were still breastfeeding at six months and those who were not.

#### Partner support

Perceived partner support was assessed using five items from the Self-Administered Questionnaire for the mother in the Québec Longitudinal Study of Child Development (QLSCD) [41]. These items measured different dimensions of partner support, including assistance with household chores, baby care, emotional support during distress, and support when feeling overwhelmed. Each item was rated on a 10-point Likert scale, ranging from 1 (not at all) to 10 (completely). A mean score of the five items was calculated to derive an overall perceived partner support score at each time point (3, 12, and 24 months), with scores ranging from 1 to 10. Participants were then categorized into two groups based on the sample mean: those scoring below the mean (perceived lower support) and those scoring at or above the mean (perceived higher support).

### Statistical analysis

After data were initially screened for missing values and univariate and multivariate normality, descriptive analyses were conducted using SPSS software (version 29; IBM Corp., 2023). Latent growth curve models (LGCM) were then estimated using Mplus software (version 8.1; Muthen and Muthen, 2012–2020). Latent growth curve models (LGCM) were used to model initial levels of, and changes in, parenting dimensions over time. LGCM captures individual differences in development longitudinally by using structural equation modeling to estimate the mean intercept (starting point) and slope (rate of change) of individuals based on their observed scores on multiple indicators of a specific construct of interest. Given the scores on the observed variables, maximum likelihood estimates were used to find the most likely values of the unobserved latent growth parameters. The mean latent intercept and slope were then used to describe the shape of the average growth curve [42].

To estimate model fit, we used the following criteria: the comparative fit index (CFI) [43] Tucker–Lewis Index (TLI; [44]), which should be greater than 0.90 [44], the root mean square error of approximation [45], with values less than 0.06, and the standardized root mean square residual (SRMR), with values less than 0.08 representing good fit. Although sensitive to sample size, the chi-square value was also considered, with a non-significant chi-square or no greater than three times the degrees of freedom indicate good model fit [43, 46].

As suggested by Preacher et al. [47], data were analyzed using a model-building approach in which a series of unconditional models (individual latent growth models without covariates) were fit to first model change over time in each parenting construct prior to examining bidirectional associations between parenting and depression variables.

To test potential moderating effects of breastfeeding and partner support on associations between maternal depression and parenting behaviours, a series of multiple-group LGCM were conducted. This approach is valuable for modeling growth trajectories and studying how growth processes may differ across the observed groups [48]. We set all paths in the model to be free and then constrained one path at a time to be equal across groups [49, 50]. To assess the impact of this equality constraint, we used the chi-square difference test. Applying this research method, we assessed the moderating effect of the total duration of breastfeeding and partner support separately on the association between MDS and parenting. Finally, mothers’ education, family income, and ethnicity were also entered as control variables in all conditional models.

## Results

### Descriptive analyses

Table 1 presents descriptive statistics for MDS and parenting dimensions at each time point. Correlations among variables, including demographics, are presented in Table 2. Mean scores on the maternal self-efficacy and maternal warmth scales at all measurement times were high, i.e., means > 8 with the maximum score on the scale being 10, but with significant variance around these means. At three, 12 and 24 months, 13.5%, 12.4% and 35.3% of participants, respectively, showed significant depressive symptoms (≧ 10; [51]) MDS from three to 12 months was stable over time, with a correlation of *r =* 0.55 (*p* < 0.001), making the modelling of its change across time with LGCM difficult. Thus, only the measures of maternal depressive symptomatology at three and 24 months were used, with the value at three months entered as a predictor and the value at 24 months entered as an outcome in the conditional models. Parenting variables from three to 12 months were less stable over time, allowing the modelling of intra-individual changes across the three time points using LGCMs.

**Table 2:** Intercorrelations among demographic variables, moderators, and depressive symptomatology and parenting across three time points

	1	2	3	4	5	6	7	8	9	10	11	12	13	14	15
1. Ethnicity															
2. Family income	-0.27***														
3. Education	-0.10***	-0.22***													
4. MDS 3 mo.	0.08*	-0.11***	-0.04												
5. MDS 24 mo.	-0.06	-0.07*	0.02	0.34***											
6. Mother’s SE 3 mo.	0.02	-0.03	0.12***	-0.30***	-0.19***										
7. Mother’s SE 12 mo.	-0.02	-0.01	0.05	-0.26***	-0.21***	0.63***									
8. Mother’s SE 24 mo.	0.01	-0.07	0.03	-0.21***	-0.23***	0.53***	0.62***								
9. Mother’s HR 3 mo.	0.04	0.02	-0.02	0.26***	0.17***	-0.31***	-0.25***	-0.25***							
10. Mother’s HR 12 mo.	0.03	-0.04	0.02	0.22***	0.13***	-0.22***	-0.27***	-0.19***	0.40***						
11. Mother’s HR 24 mo.	-0.03	0.01	-0.01	0.17***	0.17***	-0.23***	-0.21***	-0.21***	0.43***	-0.48***					
12. Mother’s WR 3 mo.	0.01	-0.05	0.09**	-0.16***	-0.8*	0.52***	0.40***	0.38***	-0.22***	-0.16***	-0.15***				
13. Mother’s WR 12 mo.	0.03	-0.04	0.02	-0.13***	-0.10**	0.37***	0.56***	0.42***	-0.14***	-0.20***	-0.18***	0.61***			
14. Mother’s WR 24 mo.	-0.01	0.01	-0.01	-0.12***	-0.10**	0.30***	0.40***	0.53***	-0.17***	-0.13***	-0.16***	0.52***	0.66***		
15. Partner support	-0.04	0.12***	-0.07**	-0.36***	-0.20***	0.23***	0.20***	0.19***	-0.21***	-0.08**	-0.05	0.14***	0.18***	0.17***	
16. Breastfeeding	0.08***	-0.04	-0.10***	0.01	-0.01	-0.06*	-0.01	-0.03	-0.5	-0.04	-0.02	-0.02	0.04	0.06	-0.04

Note. *p< 0.05. **p < 0.01. ***p < 0.001; MDS = Maternal depressive symptomatology; SE = Mother’s self-efficacy; HR=Mother’s hostile-reactive behavior; WR=Mother’s warmth

### Latent growth curve analyses

#### Unconditional models for parenting practices

A series of unconditional models of parental practices were estimated to model initial levels and change in parenting practices from three to 24 months and to find an optimal solution for each outcome, modelling linear growth and freely estimated (non-linear) growth models. For parsimony, models with residual variances set to be equal between measurement times (homoscedasticity) were favored (if model fit improved). All models were centered at the first time point (three months), with the intercept representing the average of the parenting dimension under study at three months. See Table 3 for model fit statistics for all models.

**Table 3:** Unconditional model parameters for latent growth models

Model	χ^2^	df	CFI	TLI	RMSEA	90% IC	SRMR	Slope		Intercept		Correlation α- β
								Mean	Variance	Mean	Variance	
Mother’s WR	0.38	1	1.00	1.00	0.00	[0.00; 0.06]	0.00	-0,11***	0.07***	9.39***	0.37***	-0.14
Mother’s SE	1.67	2	1.00	1.00	0.00	[0.00; 0.05]	0.04	-0.15***	0.55***	8.55***	0.61***	-0.01
Mother’s HR	1.53	2	1.00	1.00	0.00	[0.00; 0.05]	0.02	1.80***	0.21	1.16***	0.55***	0.81
Mother’s WR and MDS	14.59	10	0.995	0.988	0.02	[0.00; 0.04]	0.03					
Mother’s SE and MDS	14.70	11	0.996	0.991	0.02	[0.00; 0.03]	0.02					
Mother’s HR behavior and MDS	7.74	10	1.00	1.00	0.00	[0.00; 0.02]	0.01					

Note. *p< 0.05. **p < 0.01. ***p < 0.001.MDS = Maternal depressive symptoms; SE = Mother’s self-efficacy; HR=Mother’s hostile-reactive behavior; WR=Mother’s warmth

#### Maternal self-efficacy

A linear model did not fit the data well for maternal self-efficacy (*X*^2^ = 7.13, df = 1, RMSEA = 0.07, CFI = 0.98, TLI = 0.97, SRMR = 0.02). A freely estimated model testing non-linear growth (i.e., freeing the loading for maternal self-efficacy at 12 months), resulted in good model fit (*X*^2^ = 1.67, df = 2, RMSEA = 0.00, SRMR = 0.04, CFI = 1.00, TLI = 1.00). When homoscedasticity was considered, model fit according to *X*^2^ (3.27, df = 3), RMSEA (0.01) and SRMR (0.03) worsened, and thus the previous freely estimated (non-linear) model was retained. Results showed that the mean initial level of maternal self-efficacy was 8.55 (*p* < 0.001) and that it decreased significantly across time, with the mean change from three to 24 months being − 0.15 (*p* < 0.001). The freely estimated factor loadings (0, 0.08 and 1) indicated that most of this decrease (92%) happened between 12 and 24 months. There was significant individual variability in initial levels (0.61, *p* < 0.001) as well as in changes in self-efficacy across time (0.55, *p* < 0.001).

#### Maternal warmth

For maternal warmth the linear model fit the data well (*X*^2^ = 0.37, df = 1, RMSEA = 0.00, SRMR = 0.00, CFI = 1.00, TLI = 1.00). Results showed that the initial average level of warmth was 9.39 (*p* < 0.00) and that warmth decreased linearly by -0.11 per time point (*p* < 0.001). Significant individual variability in the initial level of warmth (0.37, *p* < 0.001) and how it changed over time (0.07, *p* < 0.001) were found.

#### Hostile-reactive behavior

A linear model did not fit the data well for hostile-reactive behavior (*X*^2^ = 9.35, df = 1, RMSEA = 0.08, CFI = 0.96, TLI = 0.88, SRMR = 0.03). A freely estimated model testing non-linear growth (i.e., freeing the loading for hostile-reactive behavior at 12 months), was considered, but did not fit the data well either (*X*^2^ = 0.32, df = 1, RMSEA = 0.00, CFI = 1.00, TLI = 1.00, SRMR = 0.01). When we considered homoscedasticity with the non-linear model, model fit improved (*X*^2^ = 1.53, df = 2, RMSEA = 0.00, SRMR = 0.02, CFI = 1.00, TLI = 1.00). This significant increase justified the inclusion of homoscedasticity, with the resulting non-linear model indicating that hostile-reactive parenting increased by 1.80 (SE = 0.06, *p* < 0.001) over time, with 59% of the increase happening between three and 12 months. Analyses showed a mean initial level of 1.16 (SE = 0.04, *p* < 0.001) of the hostile-reactive behavior scale and showed that there is individual variability in the initial level (0.55, *p* < 0.001) of hostile-reactive behavior but not how it changed over time (0.21, *p =* 0.44).

### Conditional latent growth curve models

#### Maternal depressive symptomatology and parenting

**Fig. 1:**
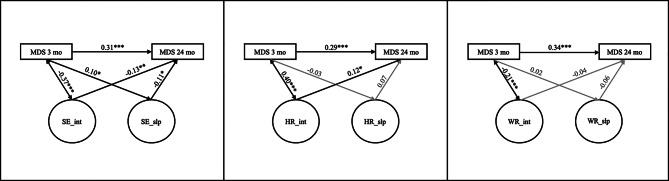
Standardized latent growth models of parenting (3–24 Months) with MDS as predictor and outcome. Notes. * *p* < 0.05 ** *p* < 0.01 *** *p* < 0.001; Standardized coefficients, controlled by mothers’ education, family income, and ethnicity; MDS = maternal depressive symptoms; SE = maternal self-efficacy; HR = hostile-reactive behavior; WR= warmth; int = intercept; slp = slope

To test the potential reciprocal association between depressive symptoms and parenting across time, maternal depressive symptomatology at three and 24 months, together with covariates (ethnicity, income and education), were entered into models with the selected latent growth factor models of the three parenting dimensions (described above; models for each parenting dimension were conducted separately). All models demonstrated a good fit (see Table 3; see Fig. 1 for graphical representation of models tested and significant associations). Higher depressive symptoms at three months were concurrently associated with lower maternal self-efficacy (*r*= -0.37, *p* < 0.001) and lower maternal warmth (*r*=-0.21, *p* < 0.001), as well as higher maternal hostile-reactive behavior (*r =* 0.39, *p* < 0.001). Depressive symptomatology at three months predicted stability in maternal self-efficacy over time (or lower decrease; β = 0.10, *p* < 0.05; as the mean of the slope is negative, higher scores represent scores closer to zero) but did not predict any change in hostile-reactive behavior or maternal warmth. Initial levels of maternal self-efficacy (β = -0.13, *p* < 0.01) and hostile-reactive behavior (β = 0.12, *p* < 0.05) at three months predicted lower and higher maternal depressive symptomatology at 24 months, respectively, while maternal warmth did not. Finally, decreases in maternal self-efficacy over time predicted higher maternal depressive symptomatology at 24 months (β = -0.11, *p* < 0.05), but not hostile-reactive behavior or maternal warmth.

### Moderation analyses

#### Breastfeeding

Two associations were found to differ significantly between mothers who breastfed and those who did not: (1) the path between depressive symptomatology at three months and the change over time in maternal warmth and (2) the path between the change over time in maternal warmth and depressive symptomatology at 24 months (Δχ2_(2)_ = 7.08, *p* < 0.05). Although these interactions reached significance because associations were in opposite directions between groups, simple slopes within each group were not significant. For maternal depressive symptomatology at three months predicting change in maternal warmth over time: associations were positive (albeit non-significant) in the breastfeeding group (β = 0.09, *p* = 0.19) but negative in the non-breastfeeding group (β= -0.17, *p* = 0.14). For change over time in maternal warmth predicting maternal depressive symptomatology at 24 months: in the breastfeeding group (β = 0.02, *p* = 0.72); in the non-breastfeeding group (β= -0.24, *p* = 0.17).

#### Partner support moderation

In all models, partner support moderated the stability of depressive symptoms across time (Δχ2_(1)_ = 7.60, *p* < 0.01). Although the stability path for depressive symptomatology at three months and 24 months was positive and significant in both groups, the stability was significantly stronger in the low support group (β = 0.36, *p* < 0.001) than in the high support group (β = 0.19, *p* < 0.01). Partner support also moderated the link between maternal depressive symptomatology and parenting dimensions. To examine these relationships more closely, we constrained different paths between maternal depressive symptomatology and parenting dimensions across the three parenting models.

First, we constrained the path between hostile-reactive behavior at three months and depressive symptomatology at 24 months. The difference between the low and high support groups was significant (Δχ2_(2)_ = 16.44, *p* < 0.001). In the low support group, hostile-reactive behaviors at three months were positively associated with depressive symptomatology at 24 months (β = 0.17, *p* < 0.05), whereas this relationship was not observed in the high support group (β = 0.04, *p =* 0.64).

Second, we constrained the path between maternal warmth over time and depressive symptomatology at 24 months. The difference between the low and high support groups was statistically significant (Δχ²_(2)_ = 8.57, *p* < 0.01). In the high support group, increases in maternal warmth over time were associated with a significant reduction in depressive symptomatology at 24 months (β = -0.14, *p* = 0.03), but not for the low support group (β = 0.03, *p* = 0.72).

Third, in addition to the stability of depression, the path between maternal self-efficacy over time (slope) and depressive symptomatology at 24 months differed significantly by partner support (Δχ²_(2)_ = 6.52, *p* < 0.05). In the low support group, a decrease in maternal self-efficacy over time was significantly related to higher depressive symptomatology at 24 months (β=-0.16, *p* < 0.01), but was not significant in the high support group (β=-0.04, *p* = 0.55). The path between maternal self-efficacy at three months and maternal depressive symptomatology at 24 months also differed significantly (Δχ²_(3)_ = 9.15, *p* < 0.05). In the low support group, lower self-efficacy at three months was significantly associated with higher depressive symptomatology at 24 months (β=-0.13, *p* < 0.05), but not in the high support group (β= -0.09, *p =* 0.15).

## Discussion

This study addresses a significant gap in the literature by analyzing the bidirectional and transactional longitudinal associations between MDS and parenting from the third to the 24th month postpartum, while exploring breastfeeding and partner support may buffer these associations. The goal was to enhance our understanding of how depressive symptoms can predict a mother’s positive and negative parenting, as well as how early parenting dimensions shape future depressive symptoms.

Our findings align with prior research indicating that maternal self-efficacy remains stable in early infancy but can decline as children grow [52]. The observed decline in self-efficacy between 12 and 24 months in our sample may stem from increasing child autonomy and behavioral challenges, which can heighten parenting stress and uncertainty [53]. Maternal warmth also declined linearly, reflecting shifts in parenting as children develop. Conversely, hostile-reactive behaviors increased, consistent with findings reported by Pierce et al. [52], of a marked increase from infancy to toddlerhood. This shift may reflect children’s growing independence and challenging behaviors during the “terrible twos”, which elicit more reactive responses leading to a cycle of coercive interaction [52, 54]. Rising hostile-reactive parenting may reinforce maternal self-doubt, contributing to declines in both self-efficacy and warmth, highlighting the need for tailored interventions to support mothers.

Furthermore, maternal depressive symptoms at three months were concurrently associated with lower maternal warmth and self-efficacy, and greater hostile-reactive behavior. This finding adds to prior research demonstrating that maternal depression is associated with later negative and positive parenting dimensions [13, 17, 55]. Moreover, we found evidence of transactional associations between MDS and self-efficacy over time. Higher depressive symptoms were associated with lower self-efficacy concurrently at three months and predicted its persistence at low levels over time. Conversely, low and declining self-efficacy over time was associated with increased depressive symptoms at 24 months. This bidirectional and transactional pattern is consistent with previous literature [17, 18] and highlights how MDS reduced mothers’ confidence in their caregiving abilities, which, in turn, contributes to worsening depressive symptoms later. Supporting mothers in maintaining their self-efficacy during the postpartum period may therefore be critical for reducing the risk of long-term depressive symptoms.

Higher hostile-reactive behavior at three months was also predictive of higher depressive symptoms at 24 months, consistent with findings reported by Lagacé-Séguinand d’Entremont [56], whereby less-than-optimal parenting may contribute to maternal depression through learned helplessness or hopelessness. Mothers who engage in early hostile-reactive behaviors may feel trapped in a cycle of ineffective parenting, reinforcing negative expectations about their ability to improve, ultimately increasing their risk of depression. Addressing early parenting difficulties may help prevent prolonged maternal depression by targeting both parenting competence and frustration.

Although breastfeeding moderated the relationship between depressive symptoms and warmth, its effects were not significant for self-efficacy or hostile-reactive behavior. This suggests that while breastfeeding may have general benefits, it might not directly buffer the effects of depressive symptoms on all parenting dimensions. Several factors may account for this limited moderating role. As noted in the introduction, breastfeeding experiences are not uniformly positive, they can be both a source of wellbeing and a source of distress depending on how initiation and continuation unfold [23, 24]. A binary variable based on duration at six months may have been too coarse to detect more nuanced effects, particularly those dependent on the quality or subjective experience of breastfeeding, dimensions more proximally linked to parenting outcomes [22]. This is consistent with Jacques et al. [57], who found that associations between breastfeeding and child outcomes differed meaningfully across duration groups, with effects emerging only at three months or more, suggesting that threshold effects may be obscured by binary operationalization. Also, the well-documented bidirectional relationship between MDS and early breastfeeding cessation [21, 24] may have introduced confounding, as mothers with higher depressive symptoms are more likely to stop breastfeeding early, potentially rendering comparison groups unequal on baseline depression severity. Future research should consider more nuanced measures of breastfeeding experience, including its quality and subjective meaning, to better capture its potential moderating role in the MDS–parenting relationship.

However, partner support played an important moderating role. Results showed that partner support may buffer the prospective association between MDS at 3 and 24 months, with that stability of depressive symptoms being lower for mothers with high partner support. Results also indicated that initial hostile-reactive behavior predicted higher depressive symptoms at 24 months, but only in mothers with low partner support. Similarly, a decline in self-efficacy over time was associated with higher depressive symptoms at 24 months, again only in the low support group. This aligns with previous research indicating that partner support is crucial for maternal well-being and parenting Brazeau et al. [30]. Several mechanisms may account for this protective role. Partner support may facilitate emotional regulation in mothers experiencing depressive symptoms, reducing the cognitive and affective load associated with parenting demands and thereby limiting the escalation of hostile-reactive behaviors [28, 58]. Additionally, practical support through shared caregiving may reduce maternal fatigue and create space for more consistent and responsive parenting, conditions under which self-efficacy may be more readily maintained [31, 59]. Finally, the presence of a supportive partner may serve as a broader relational buffer, attenuating the impact of depressive symptoms on the quality of the parent-child relationship [60]. Taken together, these mechanisms suggest that partner support operates not only at the level of emotional well-being, but also through the relational and practical conditions that enable more effective parenting.

### Strengths and limitations

Our study’s strengths include a large-scale longitudinal design, well-validated measures and a latent growth statistical approach to disentangle longitudinal and transactional associations. However, findings must be interpreted within the context of several limitations. First, the study focused exclusively on three self-reported parenting factors: warmth, hostile-reactive behavior, and self-efficacy. This may overlook other relevant parenting practices that could predict the dynamics of maternal depression and parenting. Second, we did not account for challenging behaviors in children, which could be a significant predictor or parenting outcomes and may have influenced our results. Third, the reliance on self-report measures for depressive symptomatology, parenting behaviors and perceived partner support may have inflated some relationships due to shared method variance. Social desirability bias can distort parents’ reports of their behaviors toward their children and their relationship satisfaction [61, 62]. Future studies should consider incorporating naturalistic observations of family interactions to accurately assess these dynamics. Fourth, our sample exhibited a higher overall socioeconomic status than the general Quebec population, which may limit the generalizability of the findings to more diverse populations. Finally, the high loss-to-follow-up rate (46% at 24 months) may have introduced bias, potentially limiting the generalizability of our findings. While we attempted to account for missing data using full information maximum likelihood (FIML), unmeasured confounders and differential attrition remain concerns for future research. Future studies should consider more diverse samples, multiple informants, and observational methods to mitigate bias and improve the understanding of these complex relationships, ultimately informing more effective interventions to support mothers experiencing depressive symptoms.

## Conclusion

This study underscores the complex bidirectional and transactional associations between maternal depressive symptoms and parenting practices over the first two years postpartum. Higher depressive symptoms at three months predicted lower maternal self-efficacy, reduced warmth, and more hostile-reactive behaviors. Moreover, depressive symptoms and self-efficacy influenced each other over time, with both early low self-efficacy and hostile-reactive behaviors predicting increased depressive symptoms at 24 months. Perceived partner support emerged as a key protective factor, buffering the stability of depressive symptoms over time and mitigating the impact of negative parenting behaviors on later depressive symptoms. In contrast, breastfeeding did not significantly moderate these relationships, suggesting that it may not directly influence all aspects of parenting. These findings emphasize the importance of early interventions targeting self-efficacy and emotional regulation, particularly for mothers with limited partner support, as these factors may be associated with the persistence of postpartum depressive symptoms and less optimal parenting practices. Supporting partner involvement and strengthening parenting efficacy during the postpartum period may represent promising avenues for promoting maternal mental health and healthier mother-child interactions.

## Data Availability

Data that supports the findings is available and will be provided by the correspondence author on a reasonable request.
